# Monochromaticity of Orientation Maps in V1 Implies Minimum Variance for Hypercolumn Size

**DOI:** 10.1186/s13408-015-0022-9

**Published:** 2015-04-08

**Authors:** Alexandre Afgoustidis

**Affiliations:** Institut de Mathématiques de Jussieu-Paris Rive Gauche, Université Paris 7 Denis Diderot, 75013 Paris, France

**Keywords:** Visual cortex, Orientation hypercolumns, Column spacing, Pinwheel density, Gaussian random fields, Kac–Rice formula

## Abstract

In the primary visual cortex of many mammals, the processing of sensory information involves recognizing stimuli orientations. The repartition of preferred orientations of neurons in some areas is remarkable: a repetitive, non-periodic, layout. This repetitive pattern is understood to be fundamental for basic non-local aspects of vision, like the perception of contours, but important questions remain about its development and function. We focus here on Gaussian Random Fields, which provide a good description of the initial stage of orientation map development and, in spite of shortcomings we will recall, a computable framework for discussing general principles underlying the geometry of mature maps. We discuss the relationship between the notion of column spacing and the structure of correlation spectra; we prove formulas for the mean value and variance of column spacing, and we use numerical analysis of exact analytic formulae to study the variance. Referring to studies by Wolf, Geisel, Kaschube, Schnabel, and coworkers, we also show that spectral thinness is not an essential ingredient to obtain a pinwheel density of *π*, whereas it appears as a signature of Euclidean symmetry. The minimum variance property associated to thin spectra could be useful for information processing, provide optimal modularity for V1 hypercolumns, and be a first step toward a mathematical definition of hypercolumns. A measurement of this property in real maps is in principle possible, and comparison with the results in our paper could help establish the role of our minimum variance hypothesis in the development process.

## Introduction

**Fig. 1:**
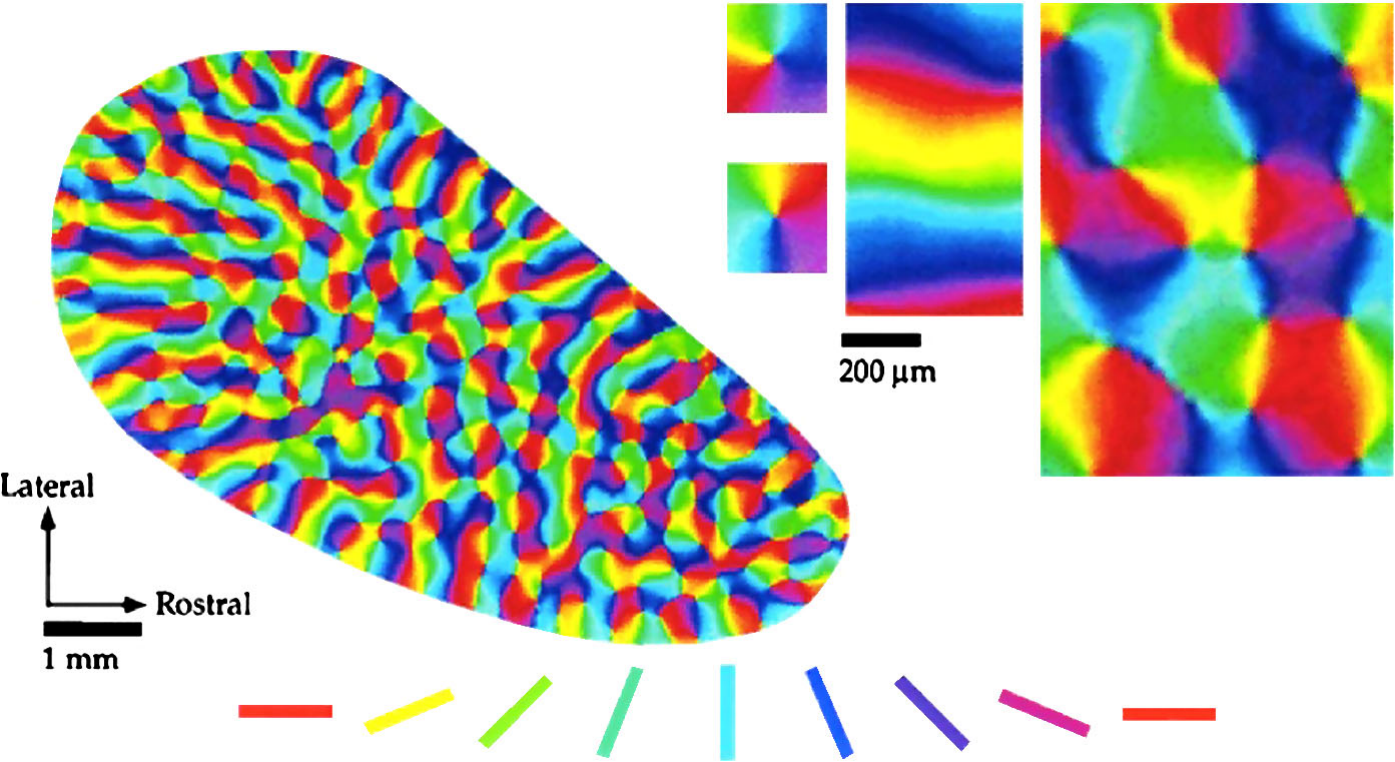
Layout of orientation preferences in the visual cortex of a tree shrew (modified from Bosking et al. [10]). Here orientation preference is *color-coded* (for instance neurons in *blue regions* are more sensitive to vertical stimuli). Maps of sensitivity to different stimulus angles were obtained by optical imaging; summing these with appropriate complex phases yields Fig. 1: see Swindale [11]. In particular, at singular points (pinwheels), all orientations meet (see *the upper right corner*); for a fine-scale experimental study of the neighbourhood of such points, see [8]

Neurons in the primary visual cortex (V1, V2) of mammals have stronger responses to stimuli that have a specific orientation [1–3]. In many species including primates and carnivores (but no rodent, even though some of them have rather elaborated vision [4, 5]), these orientation preferences are arranged in an ordered map along the cortical surface. Moving orthogonally to the cortical surface, one meets neurons with the same orientation preference; traveling along the cortical surface, however, reveals a striking arrangement in smooth, quasi-periodic maps, with singular points known as *pinwheels* where all orientations are present [6–8]; see Fig. 1. All these orientation maps look similar, even in distantly related species [5, 9]; the main difference between any two orientation preference maps (OPM) seems to be a matter of global scaling.

The common design has very precise and beautiful geometrical properties, and universal quantitative properties of these cortical maps have recently been uncovered: for instance, a density of singular points close to *π* has been observed [9, 12]; see below. However, the exact functional advantage of this geometrical arrangement in hypercolumns remains unclear [5, 11, 13–16]. What is more, the functional principles underlying the observed properties of orientation maps are still in debate; in particular, it is often thought that a pinwheel density of *π* has to do with monochromaticity (existence of a critical wavelength in the correlation spectrum) of the cortical map. The aim of this short paper is to clarify the role of the monochromaticity, or spectral thinness, condition, using the simplified mathematical framework of Gaussian Random Fields with symmetry properties. Our first few remarks (Sect. 2.1) are included for clarification purposes: we first give an intrinsic definition of the column spacing in these fields, then discuss the intervention of spectral thinness in theoretical and experimental results related to pinwheel densities. Then (Sect. 2.2) we introduce the “minimum variance” property in our title, to help discuss the quasi-periodicity in the map and to try to understand better the notion of cortical hypercolumn. In the concluding Discussion (Sect. 3), we also try to clarify the relevance of this property for the development of real maps and formulate a simple test for our hypothesis that it is indeed relevant.

Many models for the development of orientation maps have been put forward [5, 17–19]; they address such important issues as the role of self-organization, or of interactions between orientation and other parameters of the receptive profiles [14, 19–22]. In this short note, we focus on a mathematical computable framework in which geometrical properties can be discussed with full proofs, and whose quantitative properties can now be compared with those of experimental maps. While we thus put the focus on the geometry of theoretical maps rather than on the most realistic developmental scenarios, we try to relate this geometry to organizing principles, viz. information maximization and perceptual invariance, which are relevant for discussing real maps. In a mathematical setting, these principles can be enforced through explicit randomness and invariance structures.

Wolf, Geisel, Kaschube and coworkers [9, 23–25] have described a wide class of probabilistic models for the development of orientation preference maps. In all these models (and in our discussion) the cortical surface is identified with the plane $\mathbb {R}^{2}$, and the orientation preference of neurons at a point *x* is given by (half) the argument of a complex number $\mathbf{z}(x)$; one adds the important requirement that the map $x \mapsto\mathbf{z}(x)$ be continuous (this is realistic enough if the modulus $|z(x)|$ stands for something like the orientation selectivity of the neurons at *x*; see [7, 11, 26]). Pinwheel centers thus correspond to zeroes of **z**.

A starting point for describing orientation maps in these models, one which we will retain in this note, is the following general principle: we should treat **z** as a random field, so at each point *x*, the complex number $\mathbf{z}(x)$ as a random variable.

Even without considering development, it is reasonable to introduce randomness, to take into account inter-individual variability. But of the statistical properties of zero-set of general random fields, our understanding is that present-day mathematics can say very little [27]; only for very specific subclasses of random fields are precise mathematical theorems available. The most important of those is the class of *Gaussian Random fields* [27–29]—a random field **z** is Gaussian when all joint laws for $(\mathbf{z}(x_{1}), \ldots ,\mathbf {z}(x_{n})) \in \mathbb {C}^{n}$ are Gaussian random variables.

If the map **z** arises from an unknown initial state and if the development features a stochastic differential equation, taking into account activity-dependent fluctuations and noise, the Gaussian hypothesis is very natural for the early stages of visual map development (see [23, 25, 30]). In the most precise and recent development models by Wolf, Geisel, Kaschube, and others [9, 14, 31], it is, however, only the initial stage that turns out to be well represented by a Gaussian field: upon introducing long-range interactions in the integral kernel of the stochastic differential equation representing the refinement of cortical circuitry, the Gaussian character of the field must be assumed to break down when the nonlinearities become significant, and the stationary states of the dynamics which represent mature maps cannot be expected to be Gaussian states. We shall comment on this briefly in Sect. 2.1.3 and come back to it in the Discussion (Sect. 3).

**Fig. 2:**
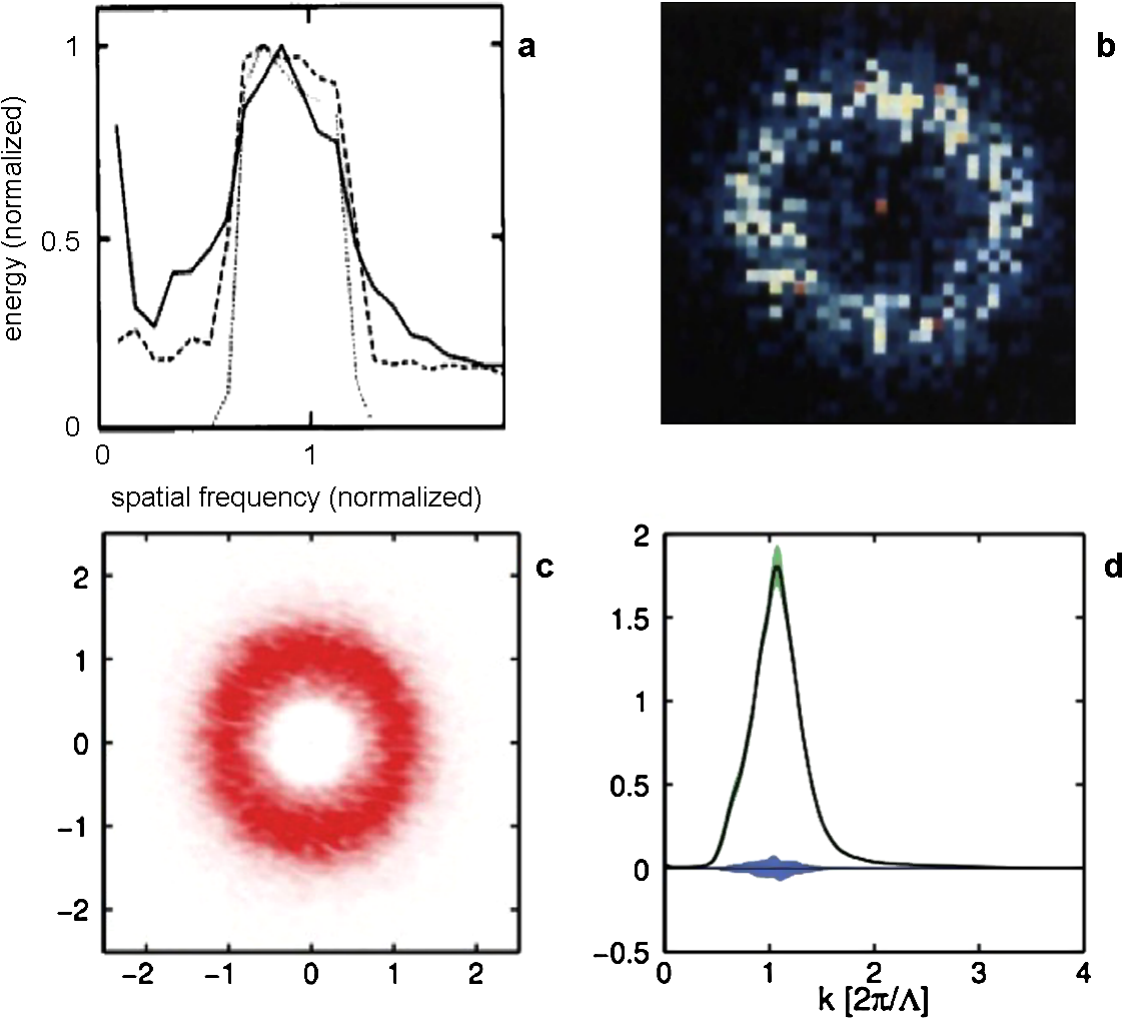
Correlation spectra of orientation maps in macaque and tree shrew V1. **a** and **b** are from Niebur and Worgotter’s 1994 paper [33]: in **a**, *the solid and dashed lines* are spectra obtained by two different methods (direct measurement of correlations and Fourier analysis) from an experimental map obtained by Blasdel in macaque monkey, the power spectrum of which is displayed on **b**. Images **c** and **d** are from Schnabel’s 2008 thesis [30, p. 104]. Methods for obtaining **c** and **d** from measurements on Tree Shrews are explained precisely by Schnabel in [30, Sects. 5.3 and 5.4]. *The green- and blue-shaded regions* code for bootstrap confidence interval and 5 % significance level, respectively. The power spectrum in **d** has standard deviation around 0.2 in the unit displayed on *the horizontal axis* and determined by the location of the maximum; the mean and quadratic wavenumbers in this spectrum are in the intervals $[1.05, 1.10]$ and $[1.18,1.23]$, respectively

In spite of this, we shall stick to the geometry of maps sampled from Gaussian Random Fields (GRFs) in our short paper. We have several reasons for doing so. A first remark is that a better understanding of maps sampled from them can be helpful in understanding the general principles underlying more realistic models, or helpful in suggesting some such principles. A second remark is that with the naked eye, it is difficult to see any difference between some maps sampled from GRFs and actual visual maps (see Fig. 2), and that there is a striking likeness between some theorems on GRFs and some properties measured in V1. A third is that precise mathematical results on GRFs can be used for testing how close this likeness is, and to make the relationship between GRFs and mature V1 maps clearer.

Wolf and Geisel add a requirement of *Euclidean invariance* on their stochastic differential equation, so that if the samples from a GRF are to be thought of as providing (early or mature) cortical maps, the field should be homogeneous (i.e. insensitive, as a random field, to a global translation $x \mapsto x + a$), isotropic (insensitive to a global rotation, $x \mapsto \bigl({\scriptsize\begin{matrix} \cos(\alpha) & -\sin(\alpha) \cr \sin(\alpha) & \cos(\alpha)\end{matrix}} \bigr) x$) and centered (insensitive to a global shift of all orientation preferences, changing the value $\mathbf{z}(x)$ at each *x* to $e^{i\theta}\mathbf{z}(x)$). Here again, looking at mature maps, geometrical invariance is a natural requirement for perceptual function; so we shall assume that the GRF **z** is centered, homogeneous, and isotropic [27, 32]. Note that of course, this invariance requirement cannot be formulated in a non-probabilistic setting (a deterministic map from $\mathbb {R}^{2}$ to ℂ cannot be homogeneous without being constant).

It actually turns out that these two mathematical constraints (Gaussian field statistics and symmetry properties) are strong enough to generate realistic-looking maps, with global quasi-periodicity. Quite strikingly, it has been observed [30, 33] that *one needs only add a spectral thinness condition* to obtain maps that seem to have the right qualitative (a hyper columnar, quasi-periodic organization) *and quantitative* properties (a value of *π* for pinwheel density). These mathematical features stand out among theoretical models for orientation maps as producing a nice quasi-periodicity, with roughly repetitive “hypercolumns” of about the same size that have the same structure, as opposed to a strictly periodic crystal-like arrangement (see [14, 21], compare [34, 35]). The aim of this short note is to clarify the importance of this spectral thinness condition for getting a quasi-periodic “hypercolumnar” arrangement on the one hand, a pinwheel density of *π* on the other.

Before we give results about homogeneous and isotropic GRFs, let us mention that the quantitative properties of the common design which have been observed by Kaschube et al. [9] also include mean values for three kinds of nearest neighbour distance and for two parameters representing the variability of the pinwheel density as a function of subregion size; evaluating these mean values in the mathematical setting of random fields, even in the oversimplified case of invariant GRFs, is a difficult mathematical problem which is beyond the author’s strengths at present. So in this short note, we shall focus on the existence of a precise hypercolumn size and a well-defined pinwheel density in the common design, and refrain from examining the other important statistics.

## Results

### Two Remarks on Gaussian Random Fields with Euclidean Symmetry

#### Preliminaries on Correlation Spectra

Let us first formulate the spectral thinness condition more precisely: in an invariant GRF, the correlation $C(x,y)$ between orientations at *x* and *y* depends only on $\Vert x-y\Vert $. Let us turn to its Fourier transform, or rather to the Fourier components of the map $\varGamma\,\colon \mathbb {R}^{2} \rightarrow \mathbb {C}$ such that $C(x,y) = \varGamma(x -y)$. For an invariant Gaussian field, specifying *Γ* does determine the field; what is more, there is a unique measure *P* on $\mathbb {R}^{+}$ such that

1 $$ \varGamma(\tau) = \int_{R>0} \varGamma_{R}(\tau) \,dP(R), $$

where, for fixed $R>0$, the map $\varGamma_{R}$ is^1^$\tau\mapsto\int_{\mathbb {S}^{1}} e^{i{R}\vec{u} \cdot\tau}\, d\vec{u}$.

Now, correlations on real cortical maps can be measured and the spectrum of *Γ* can be inferred [30]; data obtained by optical imaging reveals that the spectral measure *P* is concentrated on an annulus ([30, p. 100], see also [33]): this means that there is a *dominant wavelength*$\varLambda_{0}$, such that the measure *P* concentrates around $R_{0} = \frac{2\pi}{\varLambda_{0}}$.

Correlation spectra of real V1 maps, first discussed in [33], have been measured precisely by Schnabel in tree shrews [30] (see [30, p. 104], Fig. 5.6(d) is reproduced in Fig. 2 below). The spectral measure *P* has a nicely peaked shape, and the very clear location of the peak is used as the dominant wavelength *Λ*; see Fig. 2. From Schnabel’s data we evaluate the standard deviation in *P* to be about 0.2*Λ* (caution: here *P* is a real correlation spectrum, not the spectral density of a GRF).

**Fig. 3:**
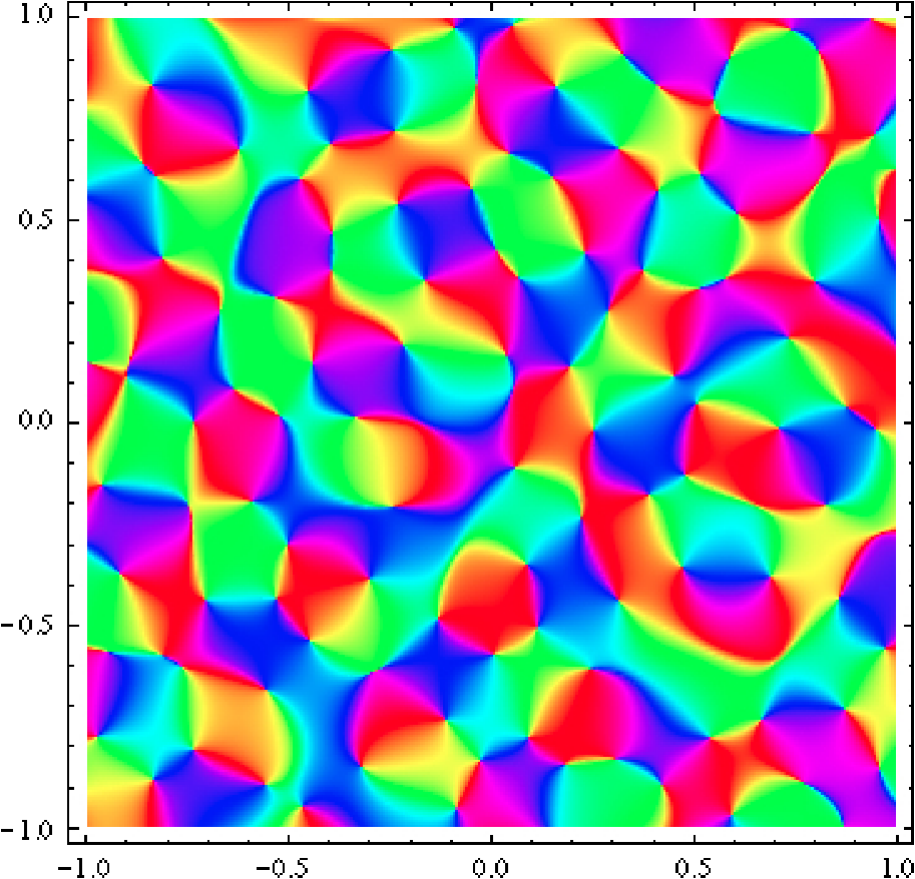
Computer-generated map, sampled from a monochromatic field. This figure shows an orientation map which we have drawn from a simulated Invariant Gaussian Random Field with circular power spectrum. We used 100 plane waves with frequency vectors at the vertices of a regular polygon inscribed in a circle, and random Gaussian weights (see the Appendix); with respect to the unit of length displayed on the *x*- and *y*-*axes*, the wavelength of the generating plane waves is $1/3$

Although this is far from being an infinitely thin spectrum, it is not absurd to look at the extreme situation where we impose the spectral thinness to be zero. Figure 3 shows a map sampled from a *monochromatic* invariant GRF, in which *Γ* is one of the maps $\varGamma_{R}$ of the previous paragraph, in other words the inverse Fourier transform of the Dirac distribution $\delta(R -R_{0})$ on a circle: monochromatic (or almost monochromatic) invariant GRFs yield quite realistic-looking maps, at least to the naked eye.

This thinness hypothesis certainly has to do with the existence of a precise scale in the map, that is, with the “hyper columnar” organization. In all existing theoretical studies that we know of, spectral thinness is introduced a priori into the equations precisely in order to obtain a repetitive pattern in the model orientation maps. For instance, in the very successful long-range interaction model of Wolf et al. [9, 31], the linear part of the stochastic differential equation for map development features a Swift–Hohenberg operator in which a characteristic wavelength is imposed. The “typical spacing” between iso-orientation domains is then defined as that which corresponds to the mean wavenumber in the power spectrum:

2 $$ \frac{2\pi}{\varLambda_{\mathrm{mean}}}:= \int k\,dP(k) . $$

#### Mean Column Spacing in Invariant Gaussian Fields

It is reasonable, both intuitively and practically, to expect that $\varLambda_{\mathrm{mean}}$ gives the mean local period between iso-orientation domains. For reasonable bell-shaped power spectra, $\varLambda_{\mathrm{mean}}$ is in addition quite close to the location of the peak in the spectrum, which very obviously corresponds to the “dominant frequency” in the power spectrum and is quite straightforward to measure. But from a mathematical point of view, there is a paradox here.

For Gaussian fields, it is natural to try to clarify this and write down an intrinsic definition of the mean column spacing in terms of the probabilistic structure of the field. The paradox is that then the natural scale to use turns out to be different from $\varLambda_{\mathrm{mean}}$, and the difference is appreciable in measured spectra. We are going to show presently that in an invariant Gaussian random field, the typical spacing turns out to be the wavelength $\varLambda_{\mathrm{sq}}$ corresponding to the quadratic mean wavenumber:

3 $$ \frac{2\pi}{\varLambda_{\mathrm{sq}}}:= \sqrt{\int k^{2}\,dP(k)}, $$

which coincides with $\varLambda_{\mathrm{mean}}$ if and only if the field is monochromatic.

Using Schnabel’s data to evaluate the corresponding wavelengths in real maps, we find that the quotient between $\varLambda _{\mathrm{sq}}$ and $\varLambda_{\mathrm{mean}}$ is about 1.1, and they are within 15 % of each other. So using one rather than the other does have an importance.

To justify our claim that $\varLambda_{\mathrm{sq}}$ is a good intrinsic way to define the column spacing in an invariant Gaussian field, let us consider a fixed value of orientation, say the vertical. Let us draw any line *D* on the plane and look for places on *D* where this orientation is represented, which means that the real part of **z** vanishes. Now if **z** is an Euclidean-invariant standard Gaussian field, $\mathfrak{Re}(\mathbf{z})|_{D}$ is a translation-invariant Gaussian field on the real line *D*. From the celebrated formula of Kac and Rice we can then deduce the typical spacing between its zeroes, and this yields the following theorem.

##### Result 1

*Pick any line segmentJof lengthℓon the plane and any orientation*$\theta_{0} \in \mathbb {S}^{1}$. *Write*$\mathcal {N}_{J,\theta_{0}}$*for the random variable recording the number of points onJwhere the Gaussian field***z***provides an orientation*$\theta_{0}$. *Then*

$$\mathbb{E}[ \mathcal{N}_{J,\theta_{0}} ] = \frac{\ell}{\varLambda _{\mathrm{sq}}}. $$

Indeed, let us write *Φ* for $\mathfrak{Re}(\mathbf{z})|_{D}$, viewed as a stationary Gaussian field on the real line, *G* for its covariance function, and $\mathcal{G}$ for the covariance function of $\mathfrak{Re}(\mathbf{z})$ viewed as a homogeneous and isotropic random field on $\mathbb {R}^{2}$. The arguments leading up to the statement of Result 1 and the Kac–Rice formula which is recalled in the Appendix prove that $\mathbb{E}[ \mathcal{N}_{J,\theta_{0}} ] = \ell\cdot\frac{\sqrt {\lambda} }{\pi}$, where $\lambda= \mathbb{E} [\varPhi'(0)^{2} ]$. But $\mathbb{E} [\varPhi'(0)^{2} ] = \partial _{x_{1}} \partial_{x_{2}} \mathbb{E} [ \varPhi(x_{1}) \varPhi(x_{2}) ] |_{x_{1} = x_{2} = 0}$, and this is $\partial_{x} \partial_{y} G(x-y) |_{x=y=0} = -G''(0)$. To complete the proof we need to calculate this.

Now, in view of the Euclidean invariance of $\mathfrak {Re}(\mathbf{z})$, we know that $G''(0)$ is half the value of $\Delta \mathcal{G}$ at zero. To evaluate this quantity, we use the spectral decomposition of $\mathcal{G}$: it reads $\mathcal{G} = \int_{R>0} \mathcal{G}_{R} \,dP(R)$, where $\mathcal{G}_{R}$ is the covariance function of a real-valued monochromatic invariant field on $\mathbb {R}^{2}$, hence is equal to $\frac{1}{2}\varGamma_{R}$ (recall that $\varGamma_{R}$ was defined in Eq. (1), and is real-valued). Now, $\varGamma_{R}$ satisfies the Helmholtz equation $\Delta( \varGamma_{R}) = -R^{2} \varGamma _{R}$, and in addition $\varGamma_{R}(0)$ is equal to 1, so $\mathcal {G}_{R}(0)$ is equal to $1/2$. We conclude that $G''(0)$ is equal to $- \frac{1}{4} \int_{R>0} R^{2}\,dP(R) = \frac{\pi ^{2}}{\varLambda_{\mathrm{sq}}^{2}}$. This completes the proof of Result 1.

Let us now comment on this result. It means that repetitions of $\theta_{0}$ occur in the mean every $\varLambda_{\mathrm{sq}}$. Of course this is very close to $\varLambda_{\mathrm{mean}}$ when the support of the power spectrum is contained in a thin enough annulus (if the width of such an annulus is less than a fifth of its radius, $\varLambda _{\mathrm{mean}}$ and $\varLambda_{\mathrm{sq}}$ are within 3 % of each other). But in general, it is obvious from Jensen’s inequality that $\varLambda_{\mathrm{mean}} \geq\varLambda_{\mathrm{sq}}$, with equality if and only if the field is monochromatic. In real maps, there is an appreciable difference between $\varLambda_{\mathrm{mean}}$ and $\varLambda_{\mathrm{sq}}$ as we saw.

#### Pinwheel Densities in Gaussian Fields and Real Maps

Let us turn now to pinwheel densities; we would like to comment on a beautiful theoretical finding by Wolf and Geisel and related experimental findings by Kaschube, Schnabel and others. We feel we should be very clear here and insist that this subsection is a comment on work by Wolf, Geisel, Kaschube, Schnabel and others; if we include the upcoming discussion it is to clarify the role of the spectral thinness condition in the proof of their result, and we seize the opportunity to comment on this work’s theoretical significance.

If a wavelength *Λ* is fixed, the pinwheel density $d_{\varLambda }$ in a (real or theoretical) map is the mean number of singularities in an area $\varLambda^{2}$. In the experimental studies of Kaschube et al. [9] and Schnabel [30], the wavelength used is obtained with two algorithms, one which localizes the maximum in the power spectrum, and one which averages local periods obtained by wavelet analysis. These two algorithms give approximately the same result, say $\varLambda_{\mathrm{exp}}$, and pinwheel densities are scaled relatively to this $\varLambda_{\mathrm{exp}}$: a very striking experimental result is obtained by Kaschube’s group, namely

4 $$ {d_{\varLambda_{\mathrm{exp}}} = \text{mean number of pinwheels in a region of area } \varLambda_{\mathrm{exp}}^{2}} \simeq \pi \pm2~\%. $$

On the other hand, in an *invariant* Gaussian random field, expectations for pinwheel densities may be calculated using generalizations of the formula of Kac and Rice. This calculation has been conducted by Wolf and Geisel [23, 25], Berry and Dennis [36]; recent progress on the mathematical formulation of the Kac–Rice formula makes it possible to write down new proofs [29, 37], as we shall see presently. The value of *π* occurs very encouragingly here, too:

##### Theorem

(Wolf and Geisel [25], Berry and Dennis [36]; see also [29, 37])

*Let us write*$\mathcal {P}_{\mathcal{A}}$*for the random variable recording the number of zeroes of the Gaussian field***z***in a region*$\mathcal{A}$, *and*$|\mathcal{A}|$*for the Euclidean area of*$\mathcal{A}$. *Then*

$$\mathbb{E}[ \mathcal{P}_{\mathcal{A}} ] = \frac{\pi}{\varLambda _{\mathrm{sq}}^{2}}|\mathcal{A}|. $$

We think it can be of interest for readers of this journal that we include a proof of this result here. We would like to say very clearly that the discovery of this result is due to Wolf and Geisel on the one hand, and independently to Berry and Dennis in the monochromatic case. In [29], Azaïs and Wschebor gave a mathematically complete statement of a Kac–Rice-type formula, and recently Azaïs, Wschebor and León used it (following Berry and Dennis) to give a mathematically complete proof of the above theorem, though they wrote down the details only in case **z** is monochromatic [37]. It is for the reader’s convenience and because the focus of this short note is with non-monochromatic fields that we recall their arguments here.

Azaïs and Wschebor’s theorem (Theorem 6.2 in [29]), in the particular case of a smooth reduced Gaussian field, is the following equality:

$$\mathbb{E}(\mathcal{P}_{\mathcal{A}}) = \frac {1}{2\pi}\int _{\mathcal{A}} \mathbb{E} \bigl\{ \bigl\vert \operatorname{det} d \mathbf{z}(p) \bigr\vert | \mathbf{z}(p) = 0 \bigr\} \,dp. $$

Here the integral is with respect to Lebesgue measure on $\mathbb {R}^{2}$, and the integrand is a conditional expectation. To evaluate this, one should first note that **z** has constant variance, and an immediate consequence is that for each *p*, the random variable $\mathbf{z}(p)$ is independent from the random variable recording the value of the derivative of the real part (resp. the imaginary part) of **z** at *p*. So the random variables $|\operatorname{det} d\mathbf {z}(p) |$ and $\mathbf{z}(p)$ are actually independent at each *p*, and we can remove the conditioning in the formula. Now at each *p*, $d\mathbf{z}(p)$ is a $2\times2$ matrix whose columns, $C_{1}(p):= \bigl({\scriptsize\begin{matrix} (\partial_{x} \mathfrak{Re} (\mathbf{z}))(p) \cr (\partial_{y} \mathfrak{Re}(\mathbf{z}))(p) \end{matrix}}\bigr) $ and $C_{2}(p):= \bigl({\scriptsize\begin{matrix} (\partial_{x} \mathfrak{Im}(\mathbf{z}))(p) \cr (\partial_{y} \mathfrak{Im}(\mathbf{z}))(p) \end{matrix}}\bigr) $, are independent Gaussian vectors (see [27, Sect. 1.4 and Chap. 5]). Because **z** has Euclidean symmetry, $C_{1}(p)$ and $C_{2}(p)$ have zero mean and the same variance, say $V_{p}$, as $(\partial_{x} \mathfrak{Re}(\mathbf{z}))(p)$. But $| \operatorname{det} d\mathbf{z}(p)|$ is the area of the parallelogram generated by $C_{1}(p)$ and $C_{2}(p)$, and the “base times height” formula says this area is the product of $\Vert C_{1}(p)\Vert $ with the norm of the projection of $C_{2}(p)$ on the line orthogonal to $C_{1}(p)$. The expectation of $\Vert C_{1}(p)\Vert $, a “chi-square” random variable, is $2\sqrt{V_{p}}$ and that of the norm of the projection of $C_{2}(p)$ on any fixed line is $\sqrt{V_{p}}$; since both columns are independent, we can conclude that

$$\mathbb{E}(\mathcal{A}) = \frac{1}{\pi}\int_{\mathcal{A}} V_{p} \,dp = \frac{|\mathcal{A}|}{\pi} V_{0} $$

(the last equality is because **z** and all its derivatives are stationary fields). Now we need to evaluate $V_{0} = \mathbb{E} \{ (\partial_{x} \mathfrak{Re} \mathbf{z})(0)^{2} \}$. But this quantity already appeared in the proof of Result 1, it was labeled *λ* there. So we already proved that it is equal to $\frac{\pi^{2}}{\varLambda_{\mathrm{sq}}^{2}}$, and this concludes the proof of Wolf and Geisel’s theorem.

From this theorem Wolf, Geisel, and others deduce that $d_{\varLambda_{\mathrm{mean}}} \geq\pi$, and it is in this form that the theorem is discussed. However, we have seen that $d_{\varLambda _{\mathrm{sq}}}$, which is equal to *π* whatever the spectrum, is a rather more natural theoretical counterpart to $d_{\varLambda_{\mathrm{exp}}}$. If we drop the focus away from $\varLambda_{\mathrm{mean}}$ to bring $\varLambda_{\mathrm{sq}}$ to the front, we obtain from Result 1 the following reformulation of Wolf and Geisel’s theorem.

##### Result 2

*Write* Δ *for the typical distance between iso*-*orientation domains*, *as expressed by Result*1, *andηfor the value*$\frac{\mathbb{E}[ \mathcal{P}_{\mathcal{A}} ]}{|\mathcal {A}|}$*of the pinwheel density*. *Then*

5 $$ \eta= \frac{\pi}{\Delta^{2}}. $$

There are two simple consequences of Wolf and Geisel’s finding which we would like to bring to our reader’s attention.

The first is that the pinwheel density of *π* observed in experiments is scaled with respect to $\varLambda_{\mathrm{exp}}$, and not with respect to $\varLambda_{\mathrm{sq}}$. Using Schnabel’s data, we can evaluate the $d_{\varLambda_{\mathrm{sq}}}$ of real maps, and as $\varLambda_{\mathrm{sq}}$ is about $0.82\varLambda_{\mathrm{exp}}$ in Schnabel’s data, $d_{\varLambda_{\mathrm{sq}}}$ strongly departs from *π* in real maps. Since it would be exactly *π* in maps sampled from GRFs, one consequence of the work in [9, 25, 30] is the following.

##### Corollary

*The pinwheel density of observed mature maps is actually* incompatible *with that of maps sampled from invariant Gaussian Fields*.

This fact is quite apparent in the work by Wolf, Geisel, Kaschube, and coworkers, but since we focused on GRFs in this short note we felt it was useful to recall this as clearly as possible.

Our second remark is that in the reformulation stated as Result 2 here, there is no longer any spectral thinness condition. In other words, *when we consider maps sampled from Gaussian Random Fields, a pinwheel density ofπis a numerical signature of the fact that the field has Euclidean symmetry.* Result 2 thus shows that when one considers invariant GRFs, average pinwheel density and monochromaticity are independent features.

Because invariant GRFs have ergodicity properties, an ensemble average such as that in Result 2 can be evaluated on an individual sample map; one can thus consider a single output of the GRF **z** and proceed to quantitative measurements on it to determine whether the probability distribution of **z** has Euclidean symmetry. Very remarkable, since no single output can have Euclidean symmetry!

To conclude this subsection, let us recall that Results 1 and 2 say nothing of map ensembles that do not have Gaussian statistics, and in particular nothing of the geometry of real maps; they certainly do not mean that the definition of $\varLambda_{\mathrm{exp}}$ used in experiments is faulty, but were simply aimed at disentangling monochromaticity from other geometrical principles in the simplified setting of GRFs. To illustrate the fact that our results are not incompatible with the definition of $\varLambda_{\mathrm{exp}}$ used in experiments, let us note that of the two methods used by Kaschube et al. to determine $\varLambda_{\mathrm{exp}}$, one (the averaging of local wavelet-evaluated spacings) provides a definition of column spacing similar to that which we used in Result 1, and the other (looking for the peak in the power spectrum) gives an appreciably different result from $\varLambda_{\mathrm{sq}}$ as we recalled. The fact that Kaschube et al. observe the two algorithms to give very close results in real maps does not go against Result 1, but rather can be seen as another argument, this time Result 1-based, against GRFs representing mature maps. The measurement of the pinwheel density, Eq. (4), furthermore indicates that development seems to keep Result 2 true at the mature stage. We shall come back to this in the Discussion.

### The Variance of Column Spacings

Results 1–2 show that for Gaussian Random Fields, the existence of a pinwheel density of *π* is independent of the monochromaticity condition. We evaluated the expected value of the column spacing in an invariant GRF in Result 1, and we now turn to its variance. There are several reasons why it should be interesting to establish rigorously that spectral thinness provides a low variance.

A first one is the search for a mathematically well-defined counterpart to the statement, visually obvious, that orientation maps are “quasi-periodic”. Most mathematical definitions of quasi-periodicity (like those which follow Harald Bohr [38]) are not very well suited to discussing V1 maps, and we feel that the meaning of the word is, in the case of V1 maps, well conveyed by the property we will demonstrate. While it is intuitively obvious that a “nice quasi-periodicity” should come with spectral thinness, as we shall see it is mathematically non-trivial.

A second reason to look at the variance is to try to understand better the concept of “cortical hypercolumn”, due to Hubel and Wiesel, which is crucial to discussions of the functional architecture of V1. Neurons in V1 are sensitive to a variety of local features of the visual scene, and a hypercolumn gathers neurons whose receptive profiles span the possible local features (note that there is no well-defined division of V1 in hypercolumns, but an infinity of possible partitionings). In studies related to the local geometry of V1 maps, once a definition for the column spacing *Λ* has been chosen, one is led (as in [9, 24, 39]) to define the area of a hypercolumn as $\varLambda^{2}$. Here we put the focus on the orientation map only; but even then is thus legitimate to wonder whether in a domain of area $\varLambda^{2}$, each orientation is represented at least once. Note that a value of *π* for the pinwheel density can guarantee this if one establishes that the density also has a small variance; here, however, we are not going to evaluate this variance, which is possible in principle [37] but not easy, and we simply focus on column spacing. This is a first step in trying to check that the internal structure of domains with area $\varLambda^{2}$ is somewhat constant, as suggested by the available results on pinwheel density.

Let us add that from the point of view of information processing, it is not unnatural to expect a low variance for hypercolumn size. It is known that the behavior of many neurons in the central nervous system depends on the statistical properties in the distributions of spikes reaching them, and not merely on the average activity. These statistical characteristics depend on physiology of course, but also on the information being vehicled. Now, vision is an active process; the eye moves ceaselessly and a given object or contour is processed by many regions of V1 in a relatively short time. For a neuron receiving inputs from V1, a low variance for hypercolumn size should help make the distribution of received information more uniform (with minimum bias for a given orientation). This would be in harmony with a general principle at work in the central nervous system, that of maximizing mutual information, which on the sensory side corresponds to a maximum of discrimination (and Fisher information; see [40]) and on the motor side to what has been called the “minimum variance principle”, for instance in the study of ocular saccades or arm movements [41].

So we will now consider the variance $\mathbb{V} [ \mathcal{N}_{J,\theta_{0}}]$ of the previous random variable. We will show that it reaches a minimum when the spectrum is a pure circle. Now, evaluating this variance is surprisingly difficult, even though there is an explicit formula, namely the following.

#### Theorem

(Cramer and Leadbetter; see [42])

*In the setting of Result*1, *write*$G\,\colon \mathbb {R}\rightarrow \mathbb {R}$*for the covariance function of*$\mathfrak{Re}(\mathbf{z})|_{D}$*and*$M_{33}(\tau)$, $M_{44}(\tau)$*the cofactors of the*$(3,3)$*and*$(3,4)$*entries in the matrix*

$$\begin{pmatrix} 1 & G(\tau) & 0 & G'(\tau) \\ G(\tau) & 1 & - G'(\tau) & 0 \\ 0 & -G'(\tau) & -G''(0) & -G''(\tau) \\ G'(\tau) & 0 & -G''(\tau) & -G''(0) \end{pmatrix} . $$

*Then*

6 $$\begin{aligned} &\mathbb{V}[ \mathcal{N}_{J,\theta _{0}} ] \\ &\quad= \frac{\pi\ell}{\varLambda_{\mathrm{sq}}} - \biggl( \frac{\pi\ell }{\varLambda_{\mathrm{sq}}} \biggr)^{2} \\ &\quad\quad{}+ \Biggl(\frac{2}{\pi^{2}} \int _{0}^{\ell} (\ell- \tau) \frac{\sqrt{M_{33}(\tau)^{2} - M_{34}(\tau)^{2}}}{(1 - G(\tau )^{2})^{3/2}} \\ &\quad\quad {}\times \biggl[ 1 + \frac{M_{34}(\tau )}{\sqrt{M_{33}(\tau)^{2} - M_{34}(\tau)^{2}}} \arctan \biggl( \frac {M_{34}(\tau)}{\sqrt{M_{33}(\tau)^{2} - M_{34}(\tau)^{2}}} \biggr) \biggr] \,d\tau\Biggr). \end{aligned}$$

Recall here that

7 $$ G(\tau) = \frac{1}{4\pi} \int_{R>0} \biggl( \int_{0}^{2\pi} \cos\bigl({R}\tau\cos(\vartheta)\bigr) \,d\vartheta \biggr) P(R)\, dR; $$

this $G(\tau)$ is an oscillatory integral which involves Bessel-like functions with different parameters, and the formula for $\mathbb{V}[ \mathcal{N}_{J,\theta_{0}} ]$ features quite complicated expressions using the first and second derivatives of this integral, with a global integration on top of this; so any analytical understanding of formula (6) seems out of reach! But we can check numerically that it does attest to monochromatic fields having minimum variance.

We used Mathematica to evaluate variances of invariant GRFs, using the formulae in the theorem of Cramer and Leadbetter’s. This needed some care: to evaluate $\mathbb{V}[ \mathcal{N}_{J,\theta_{0}}] $, we had to perform numerical integration on an expression involving derivatives of the correlation function *G*, itself a parameter-dependent integral which cannot be reduced to simpler functions of the parameter. This kind of numerical evaluation is rather delicate to perform precisely, especially if there are oscillations in the integral as is the case here—the result can then be very highly dependent on the sampling strategy—and if there are multiple operations to be performed on the outputs of these integrals—the calculations of derivatives and second derivatives of the numerically evaluated *G*, and the multiple divisions, might propagate the errors quite erratically.

**Fig. 4:**
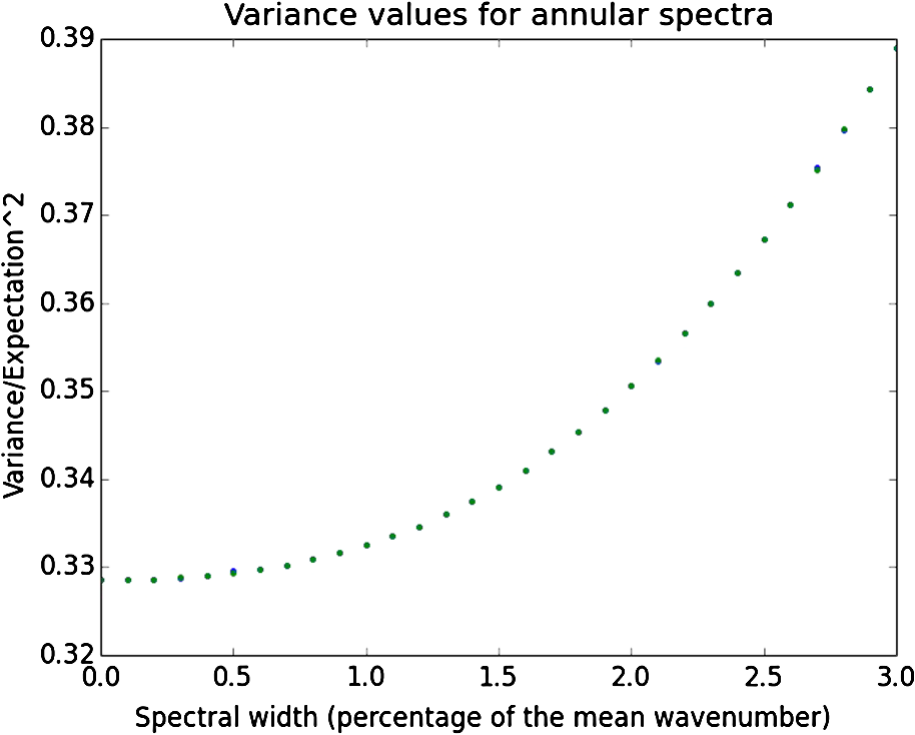
Variance is a decreasing function of spectral thinness. This is a plot of the variance of the random variable recording the number of times a given orientation is present on a straight line segment of fixed length. We considered here Invariant Gaussian Random Fields with uniform power spectra, and plotted the variance as a function of spectral width. For each percentage of the mean wavenumber, we displayed two outputs to give an idea of the attained precision. A low value for variance, here expressed in the unit given by the square of the expectation, corresponds to a field whose hypercolumns have relatively constant size across the resulting orientation map; the results displayed here show that a very regular hypercolumnar organization is quite compatible with stochastic modeling, and is a direct consequence of the spectral thinness condition found in models. Moreover, the horizontal slope at zero shows that as regards the global properties of quasi-periodic maps, there is very little difference between a theoretically ideal monochromaticity and a more realistic (and more model-independent) spectral thinness

In order to keep the numerical errors from masking the “exact” effect of thickening the spectrum, we forced the software to optimize its calculation strategy (adaptive Monte-Carlo integration), detecting oscillations in the integrand and adapting the sampling requirements, and we extended evaluation time beyond the usual limits (by dropping the in-built restrictions on the recursion depths). When the difference between successive evaluations was tamed, this yielded the variance curve displayed on Fig. 4.

Note that the drawn variances correspond to fields with very slightly different spacings $\varLambda_{\mathrm{sq}}$; however, it is easy to check numerically that for every spectrum considered here, the variance of a monochromatic field with wavelength $\varLambda_{\mathrm{sq}}$ is inferior to the variance drawn on Fig. 4.

Numerical evaluations also show that at a fixed spectral width, using few circles to build the field (i.e. introducing several characteristic wavelengths in the map) leads to a higher variance than simulating a uniform spectral distribution. To see this, we first evaluated $\mathbb{V}[ \mathcal{N}_{J,\theta_{0}}] $ for an invariant GRF whose spectrum gathered three circles of radii $R_{\mathrm{inf}}$, $R_{\mathrm{sup}}$ and $R_{\mathrm{mean}} = 10.95$ in the fixed arbitrary unit, then spanned the interval between $R_{\mathrm{inf}}$ and $R_{\mathrm{sup}}$ with more and more circles, using spectra with $2N+1$ circles of radii $R_{\mathrm{mean}} + 0.95\frac{i}{N}$. We observed $\mathbb{V}[ \mathcal{N}_{J,\theta_{0}}] $ to decrease with *N* in that case, and the existence of a limit value. From Riemann’s definition of the integral, we see that this value is that which corresponds to a spectrum uniformly distributed in the annulus delimited by $R_{\mathrm{inf}}$ and $R_{\mathrm{sup}}$. To keep the evaluation time reasonable (it is roughly quadratic in *N*), we kept the value $N = 18$ for the evaluations whose results are shown on Fig. 3, and which are close to the observed limit values. We should also add here that we observed higher values for variance when using smooth spectra with several dominant wavelengths.

This is another argument for monochromaticity yielding minimum variance. Since the space of possible spectra with a fixed support is infinite-dimensional, our numerical experiments cannot explore it all. But we feel justified in stating the following numerical results on quasi-periodicity in orientation maps sampled from invariant Gaussian fields.

#### Result 3

(i) *For uniform spectra*, *variance increases with the width of the supporting annulus*.
(ii) *For a given spectral width*, *dominance of a single wavelength seems to yield minimum variance*. *Introducing more than one critical wavelength in the spectrum systematically increases nonuniformity in the typical size of hypercolumns*.

Result 3 proves that sharp dominance of a single wavelength is the best way to obtain minimum variance. What is more, the horizontal slope at zero in Fig. 3 means that fields which are close to monochromatic have much the same quasi-periodicity properties as monochromatic invariant fields. This is quite welcome in view of Schnabel’s results: of course we cannot expect actual monochromaticity in real OPMs, but clear dominance of a wavelength is much more reasonable biologically. A more theoretical benefit is the flexibility of invariant GRFs for modeling: a model-adapted precise formula for the power spectrum may be inserted without damage to the global, robust resemblance between the predicted OPMs and real maps [5].

These observations reinforce the hypothesis that our three informational principles (randomness structure, invariance, spectral thinness) are sufficient to reproduce quantitative observable features of real maps, though as we saw, using an invariant GRF with the most realistic spectrum does not necessarily yield a more realistic result than using a monochromatic GRF, and leads to incompatibilities with the observed mature maps. This form of universality is certainly welcome: individual maps in different animals, from different species (with different developmental scenarii) necessarily have different spectra, but general organizing principles *can* be enough to explain even quantitative observed properties.

## Discussion

In this short note we recalled that simple hypotheses on randomness, invariance, and spectral width of model orientation maps reproduce important geometrical features of real maps. Though it should not be forgotten that we worked in a simplified mathematical framework which reproduces only some aspects of the common design and whose dissemblance with real maps can be established rigorously as we recalled, we feel two new points deserve special attention: first, we showed that in the simplified setting of Gaussian Random Fields, the best mathematical quantity for explaining the local quasi-period is the quadratic mean wavenumber rather than the mean wavenumber, and pointed out that a pinwheel density of *π*, when scaled with respect to this intrinsic column spacing, is a signature of Euclidean symmetry and not of Euclidean symmetry plus spectral thinness; second, we established (through numerical analysis of an exact formula) that the variability of local quasi-periods is minimized when the standard deviation of the spectral wavelength tends to zero.

Our analysis shows that at least in the setting of Gaussian fields, realistically large spectra are compatible with a low variance; we suggest that a low variance for column spacing might be observed in real data, and perhaps also a low variance for the number of pinwheels in an area $\varLambda_{\mathrm{exp}}^{2}$. Spectral thinness is usually attributed to biological hardware in the cortex (like pre-sight propagation wavelengths in the retina or thalamus [43, 44]); this turns out to be compatible with some form of optimality in information processing.

It would also be very interesting to compare the variance of column spacings in real maps (in units of the spacing evaluated by averaging local periods) with the smallest possible value for GRFs, observed in this paper for monochromatic fields (see Fig. 4); if a lower value for variance in real maps than in monochromatic Gaussian fields is found, it would mean that cortical circuitry refinement, featuring long-range interactions, brings mature maps closer to a geometrical homogeneity of hypercolumns. This would also throw some light on the fact that as development proceeds and the probability distribution of the field turns away from that of a GRF, driven by activity-dependent shaping, the column spacing obtained by averaging local periods seems to come closer to the wavelength associated to the mean or peak wavenumber (see [9, supplementary material, p. 5]) than it is in GRFs. It is then remarkable that development should maintain the value of *π* for pinwheel density when scaled with respect to the current value of column spacing, keeping Result 2 valid over time (of course the density seems to move if one does not change the definition of column spacing over time, but the best-suited quantity for measuring column spacing seems to change). Perhaps this also has a benefit for areas receiving inputs from V1, keeping their tuning with the pinwheel subsystem (which seems to have an independent interest for information processing; see [45]) stable.

## Appendix

### A.1 Sampling from Monochromatic Invariant Random Fields

In the main text, we defined monochromatic invariant Gaussian random fields through their correlation functions, and we studied the difference between monochromatic invariant fields and general invariant Gaussian fields. On Fig. 2 we displayed an OPM sampled from a monochromatic invariant Gaussian field, but we did not say how the drawn object was built from its correlation function. We provide some details in this subsection.

Recall that the covariance function of a monochromatic invariant Gaussian random field with correlation wavelength *Λ* is provided by the inverse Fourier transform of the Dirac distribution on a circle, that is,

$$C(x,y)=\mathbb{E} \bigl[\mathbf{z}(x) \mathbf{z}(y)^{\star}\bigr] = \varGamma (x -y) = \int_{\mathbb {S}^{1}} e^{i R u \cdot(x -y)} \,du $$

with $R = \frac{2\pi}{\varLambda}$. Now, *Γ* satisfies the Helmholtz equation $\Delta\varGamma= -R^{2} \varGamma$; from this we can easily deduce that

$$\mathbb{E} \bigl[ \bigl| \Delta\mathbf{z} + R^{2} \mathbf{z} \bigr|^{2} \bigr] $$

is identically zero. This means that any (strictly speaking, almost any) orientation map drawn from **z** satisfies itself the Helmholtz equation; thus OPMs drawn from **z** are superpositions of plane waves with wavenumber *R* and various propagation directions.

Thus, we know that there is a random Gaussian measure $d\mathbb{Z}$ on the circle which allows for describing **z** as a stochastic integral:

$$\mathbf{z}(x) = \int_{\mathbb {S}^{1}} e^{i R u \cdot x } \,d\mathbb{Z}(u). $$

Now, from the Gaussian nature of **z** and the Euclidean invariance condition, we have a simple way to describe ℤ, which we used for actual computations: if $(\zeta_{k})_{k \in \mathbb {N}^{\star}}$ is a sequence of independent standard Gaussian complex random variables, and if $u_{1}, \ldots, u_{n}$ are the complex numbers coding for the vertices of a regular *n*-gon inscribed in the unit circle, then

$$\mathbf{z}_{n}\,\colon x \mapsto\frac{1}{n} \sum _{i=1}^{n} \zeta _{i} e^{i R u_{i} \cdot x } $$

is a Gaussian random field. As *n* grows to infinity, we get random fields which are closer and closer to a monochromatic invariant Gaussian random field, and our field **z** is but the limiting field.

### A.2 Kac–Rice Formula

We derived Result 1 from the classical Kac–Rice formula, and the theorem from which we obtained Result 2 can be obtained from a suitable generalization to plane random fields (see [29, 37] and [25, 27, 36] in the main text). Here we give the precise theorem we used in the derivation of Result 1. This formula was obtained as early as 1944, though the road to a complete proof later proved sinuous; the initial motivation on Rice’s side was the study of noise in communication channels, which can be thought of as random functions of time. For modeling noise it is then reasonable to introduce Gaussian random fields defined on the real line, and if the properties of the communication channel do not change over time, to assume further that they are stationary. Rice discovered that there is a very simple formula for the mean number of times this kind of field crosses a given “noise level”; this is the

#### Classical Kac–Rice formula

*Consider a stationary Gaussian Random Field**Φ**defined on the real line*, *with smooth trajectories*; *choose a real number**u*, *and consider an interval**I**of length**ℓ**on the real line*. *Write*$\mathcal{N}_{u, I}$*for the random variable recording the number of points**x**on**I**where*$\varPhi (x) = u$; *then*

$$\mathbb{E} [ \mathcal{N}_{u, I} ] = \ell\cdot\frac{ e^{-u^{2}/2} \sqrt{\lambda} }{\pi}, $$

*where*$\lambda= \mathbb{E} [\varPhi'(0)^{2} ]$*is the second spectral moment of the field*.

For the proof of this old formula, as well as a presentation of all the features of GRFs underlying our main text, see [27, 42]. For the proof of the theorem we used for Result 2, which is much more recent, we refer to [25] and [37].

Just after Result 1, we mentioned that the comparison between $\varLambda _{\mathrm{mean}}$ and $\varLambda_{\mathrm{sq}}$ is an immediate consequence of Jensen’s inequality, so let us give its statement here. We start with a continuous probability distribution ℙ on the real line. Now whenever *φ* is a *convex*, real-valued function on ℝ, Jensen’s inequality is the fact that, for each measurable function *f*,

$${ \varphi \biggl( \int_{\mathbb {R}} f(x) \,d\mathbb{P}(x) \biggr) \leq\int_{\mathbb {R}} \varphi \bigl( f(x) \bigr) \,d\mathbb{P}(x) } $$

(compare the triangle inequality). In the main text, we used it when ℙ is the power spectrum distribution of a random field, *f* is the identity function of ℝ, and *φ* is $x \mapsto x^{2}$.

## Footnotes

1 The measure on $\mathbb {S}^{1}$ used in this formula has total mass one.

## References

[1] Hubel DH, Wiesel TN (1962). Receptive fields, binocular interaction and functional architecture in the cat’s visual cortex. J Physiol.

[2] Hubel DH, Wiesel TN (1959). Receptive fields of single neurones in the cat’s striate cortex. J Physiol.

[3] Hubel DH, Wiesel TN (1968). Receptive fields and functional architecture of monkey striate cortex. J Physiol.

[4] Van Hooser SD, Heimel JA, Nelson SB (2005). Functional cell classes and functional architecture in the early visual system of a highly visual rodent. Prog Brain Res.

[5] Kaschube M (2014). Neural maps versus salt-and-pepper organization in visual cortex. Curr Opin Neurobiol.

[6] Bonhoeffer T, Grinvald A (1993). The layout of iso-orientation domains in area 18 of cat visual cortex: optical imaging reveals a pinwheel-like organization. J Neurosci.

[7] Bonhoeffer T, Grinvald A (1991). Iso-orientation domains in cat visual cortex are arranged in pinwheel-like patterns. Nature.

[8] Ohki K, Chung S, Kara P, Hubener M, Bonhoeffer T, Reid RC (2006). Highly ordered arrangement of single neurons in orientation pinwheels. Nature.

[9] Kaschube M, Schnabel M, Lowel S, Coppola DM, White LE, Wolf F (2010). Universality in the evolution of orientation columns in the visual cortex. Science.

[10] Bosking WH, Zhang Y, Schofield B, Fitzpatrick D (1997). Orientation selectivity and the arrangement of horizontal connections in tree shrew striate cortex. J Neurosci.

[11] Swindale NV (1982). A model for the formation of orientation columns. Proc R Soc Lond B, Biol Sci.

[12] Miller KD (2010). *π* = visual cortex. Science.

[13] Yu H, Farley BJ, Jin DZ, Sur M (1995). The coordinated mapping of visual space and response features in visual cortex. Neuron.

[14] Reichl L, Heide D, Lowel S, Crowley JC, Kaschube M, Wolf F (2012). Coordinated optimization of visual cortical maps (I) symmetry-based analysis. PLoS Comput Biol.

[15] Petitot J (2008). Neurogéométrie de la vision: modeles mathematiques et physiques des architectures fonctionnelles.

[16] Barbieri D, Citti G, Sanguinetti G, Sarti A (2012). An uncertainty principle underlying the functional architecture of V1. J Physiol (Paris).

[17] Swindale NV (1996). The development of topography in the visual cortex: a review of models. Netw Comput Neural Syst.

[18] Chalupa LM, Werner JS (2004). The visual neurosciences. Vol. 1.

[19] Nauhaus I, Nielsen KJ (2014). Building maps from maps in primary visual cortex. Curr Opin Neurobiol.

[20] Swindale N, Shoham D, Grinvald A, Bonhoeffer T, Hubener M (2000). Visual cortex maps are optimized for uniform coverage. Nat Neurosci.

[21] Reichl L, Heide D, Lowel S, Crowley JC, Kaschube M (2012). Coordinated optimization of visual cortical maps (II) numerical studies. PLoS Comput Biol.

[22] Nauhaus I, Nielsen K, Disney A, Callaway E (2012). Orthogonal micro-organization of orientation and spatial frequency in primate primary visual cortex. Nat Neurosci.

[23] Wolf F, Geisel T (1998). Spontaneous pinwheel annihilation during visual development. Nature.

[24] Kaschube M, Wolf F, Geisel T, Lowel S (2002). Genetic influence on quantitative features of neocortical architecture. J Neurosci.

[25] Wolf F, Geisel T (2003). Universality in visual cortical pattern formation. J Physiol (Paris).

[26] Nauhaus I, Busse L, Carandini M, Ringach D (2008). Stimulus contrast modulates functional connectivity in visual cortex. Nat Neurosci.

[27] Adler RJ, Taylor JE (2009). Random fields and geometry.

[28] Abrahamsen P. A review of Gaussian random fields and correlation functions. 2nd ed. Oslo (Norway): Norsk Regnesentral; 1997 Apr. Report No.: 917. 64 p.

[29] Azaïs JM, Wschebor M (2009). Level sets and extrema of random processes and fields.

[30] Schnabel M. A symmetry of the visual world in the architecture of the visual cortex [PhD thesis]. [Goettingen (Germany)]: University of Goettingen; 2008.

[31] Wolf F (2005). Symmetry, multistability, and long-range interactions in brain development. Phys Rev Lett.

[32] Yaglom AM (1961). Second-order homogeneous random fields. Proceedings of the fourth Berkeley symposium on mathematical statistics and probability. Vol. 2, Contributions to probability theory.

[33] Niebur E, Worgotter F (1994). Design principles of columnar organization in visual cortex. Neural Comput.

[34] Koulakov A, Chklovskii D (2001). Orientation preference patterns in mammalian visual cortex: a wire length minimization approach. Neuron.

[35] Paik SB, Ringach D (2011). Retinal origin of orientation maps in visual cortex. Nat Neurosci.

[36] Berry MV, Dennis MR (2000). Phase singularities in isotropic random waves. Proc R Soc Lond A, Math Phys Sci.

[37] Azaïs JM, León JR, Wschebor M (2011). Rice formulae and Gaussian waves. Bernoulli.

[38] Bohr HA (1947). Almost periodic functions.

[39] Kaschube M, Schnabel M, Wolf F (2008). Self-organization and the selection of pinwheel density in visual cortical development. New J Phys.

[40] Zhang K, Sejnowski TJ (1999). Neuronal tuning: to sharpen or broaden?. Neural Comput.

[41] Harris CM, Wolpert DM (1998). Signal-dependent noise determines motor planning. Nature.

[42] Cramer H, Leadbetter MR (1967). Stationary and related stochastic processes. Sample function properties and their applications.

[43] Ernst UA, Pawelzik KR, Sahar-Pikielny C, Tsodyks MV (2001). Intracortical origin of visual maps. Nat Neurosci.

[44] Maffei L, Galli-Resta L (1990). Correlation in the discharges of neighboring rat retinal ganglion cells during prenatal life. Proc Natl Acad Sci USA.

[45] Dragoi V, Rivadulla C, Sur M (2001). Foci of orientation plasticity in visual cortex. Nature.

